# Pharmacokinetics of the nonsteroidal steroid sulphatase inhibitor 667 COUMATE and its sequestration into red blood cells in rats

**DOI:** 10.1038/sj.bjc.6602130

**Published:** 2004-08-24

**Authors:** C R Ireson, S K Chander, A Purohit, D C Parish, L W L Woo, B V L Potter, M J Reed

**Affiliations:** 1 Endocrinology and Metabolic Medicine and Sterix Ltd, Faculty of Medicine, Imperial College, St Mary's Hospital, London W2 1NY, UK; 2 Medicinal Chemistry and Sterix Ltd, Department of Pharmacy and Pharmacology, University of Bath, Claverton Down, Bath BA2 7AY, UK

**Keywords:** oestrogens, androstenediol, oestrone sulphate, steroid sulphatase, 667 COUMATE

## Abstract

Breast cancer is a major cause of mortality in Western countries and there is an urgent requirement for novel treatment strategies. The nonsteroidal sulphatase inhibitor 667 COUMATE inhibits hepatic steroid sulphatase and growth of oestrone sulphate stimulated tumours in the nitrosomethylurea-induced rat mammary model. Other compounds that contain an aryl sulphamate moiety, for example, oestrone-3-*O*-sulphamate, are sequestered into red blood cells (RBCs). The aims of this study were to determine the pharmacokinetics of 667 COUMATE and to investigate its sequestration into RBCs. We administered a single p.o. or i.v. dose (10 mg kg^−1^) of 667 COUMATE to rats and used a high-performance liquid chromatography method to measure the levels of the agent and its putative metabolites in plasma. 667 COUMATE had a bioavailability of 95% and could be detected in plasma for up to 8 h. Using two independent analytical methods, we demonstrated that 667 COUMATE is sequestered by RBCs both *ex vivo* and *in vivo.* Previous investigations have revealed that 667 COUMATE is rapidly degraded in plasma *ex vivo*. In this study, we demonstrate that 667 COUMATE is stabilised due to its sequestration into RBCs. In conclusion, the pharmacological efficacy and high oral bioavailability of 667 COUMATE may be partly a consequence of the ability of RBCs to both protect the agent from metabolic degradation and facilitate its transport to tissues. These data support the further clinical evaluation of this novel endocrine therapeutic agent.

Breast cancer is the most common form of cancer among women in the developed world (Parkin *et al*, 1999). Oestrogens are considered to be the most important hormones that influence the development and growth of breast tumours (James and Reed, 1980; Bernstein and Ross, 1993). In postmenopausal women, in whom breast cancer most frequently occurs, oestrogens are formed exclusively in peripheral tissues. There are two main pathways by which oestrogens can be synthesised in peripheral tissues, that is, the aromatase and sulphatase routes. In the aromatase pathway, androstenedione, secreted mainly by the adrenal cortex, is converted to oestrone by the aromatase enzyme complex. Much of this oestrone formed via the aromatase route can be converted to oestrone sulphate (E1S) by sulphotransferase enzymes (Strott, 2002). Plasma and tissue concentrations of E1S in humans are considerably higher than those of unconjugated oestrone and oestradiol (Noel *et al*, 1981; Pasqualini *et al*, 1996). It has been suggested that the high plasma and tissue levels of E1S may act as a reservoir for the formation of biologically active steroids by the action of steroid sulphatase (STS) (Reed *et al*, 1996). The activity of STS, the enzyme that hydrolyses E1S to oestrone, is considerably higher than that of the aromatase enzyme in breast tumours (James *et al*, 1987). The potential importance of the sulphatase pathway for the *in situ* formation of oestrogen from oestrogen sulphates was recently demonstrated in a study in which MCF-7 breast cancer cells transfected with either vector (MCF-7v) or vector containing the STS cDNA (MCF-7_STS_) were inoculated into the flanks of ovariectomised nude mice (James *et al*, 2001). The incidence of tumours in mice bearing MCF-7_STS_ cells and supplemented with oestradiol sulphate (71%) was significantly higher than in animals bearing this cell line but not supplemented with oestradiol sulphate (21%). Supplementation with oestradiol sulphate, and subsequent hepatic hydrolysis, was not sufficient to stimulate the growth of MCF-7 cells, thus demonstrating the importance of *in situ* oestrogen synthesis in supporting tumour growth. Recently, four studies have confirmed that a high level of STS mRNA expression in tumours was associated with a poor prognosis in women with breast cancer (Utsumi *et al*, 1999; Miyoshi *et al*, 2003; Suzuki *et al*, 2003; Yoshimura *et al*, 2004). In contrast, aromatase mRNA expression had no prognostic value. Since many tumours will fail to respond to endocrine therapy, or progress after a relatively short period of time, it is necessary to continue to develop new drugs for the treatment of breast cancer. A number of potent STS inhibitors have now been developed (Howarth *et al*, 1994; Reed *et al*, 1996), of which 667 COUMATE (Figure 1

**Figure 1:**
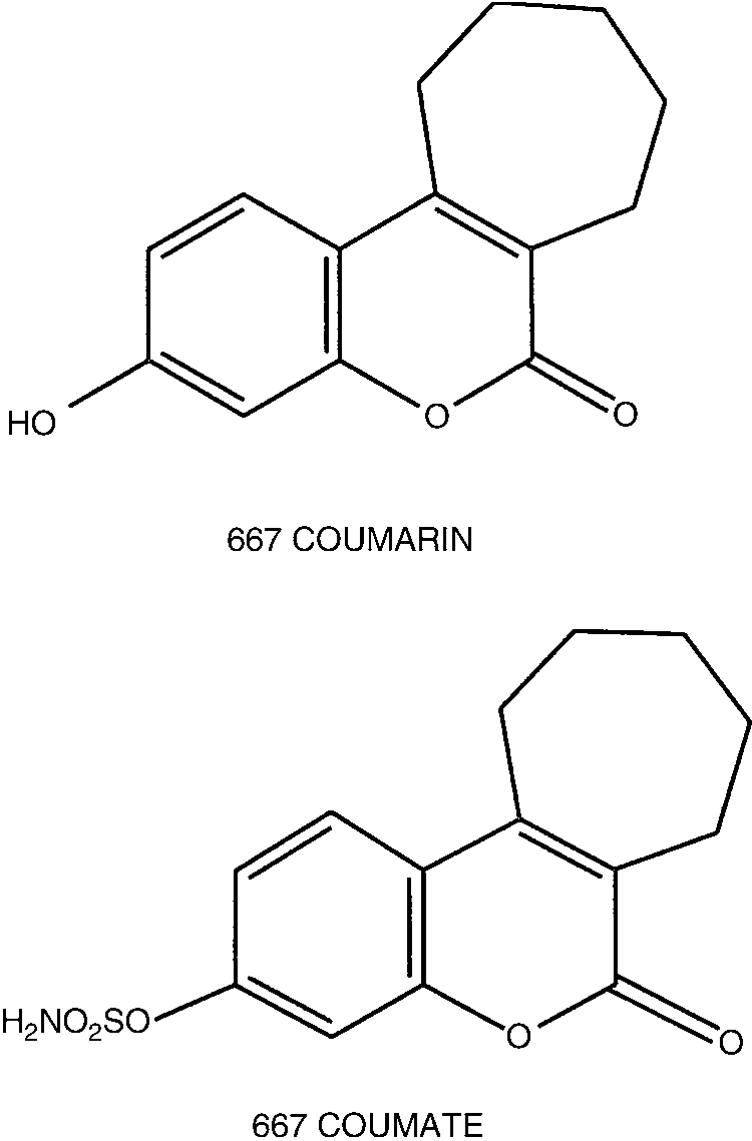
Chemical structures of 3-Hydroxy-6-oxo-8,9,10,11-tetrahydro-7*H*-cyclohepta-[c] [1] benzopyran (667 COUMARIN) and 6-oxo-8,9,10,11-tetrahydro-7*H-*cyclohepta-[c] [1] benzopyran-3-*O*-sulphamate (667 COUMATE).

) (Purohit *et al*, 2000; Woo *et al*, 2000) has recently entered a Phase I clinical trial in postmenopausal women with breast cancer.

While there is evidence for the efficacy of 667 COUMATE *in vivo* in the rat, its pharmacokinetics have not been investigated. In order to establish the pharmacokinetic parameters of 667 COUMATE, we administered a single oral (p.o.) or intravenous (i.v.) dose of the agent to female rats. In a previous study, we investigated the stability of 667 COUMATE in plasma (Ireson *et al*, 2003). When the drug was incubated with plasma, 80% of the agent was removed by 4 h. The major degradation product was 667 COUMARIN (Figure 1), which was devoid of any STS inhibitory activity. Paradoxically, a single dose of 667 COUMATE has been shown to inhibit STS *in vivo* in the rat for up to 24 h (Purohit *et al*, 2001). 667 COUMATE contains a sulphamate moiety (Figure 1). It is apparent that other agents containing this chemical group, for example, oestrone-3-*O*-sulphamate (EMATE), are sequestered into red blood cells (RBCs) after absorption in which they are able to transit the liver without first pass metabolism and inactivation (Elger *et al*, 1995, 1998). It is known that a number of sulphonamide drugs, which are structurally related to sulphamates, are also transported in RBCs (Lin *et al*, 1992). Their transport in these cells is facilitated by binding reversibly to carbonic anhydrase II (CAII), which is expressed in the cytoplasm of RBCs, via coordination of the sulphamate anion to the active site zinc atom.

We hypothesised that 667 COUMATE is also sequestered into RBCs and that this may explain, at least in part, the efficacy of the agent *in vivo* in the rat despite its modest stability *ex vivo* in plasma. Indeed, 667 COUMATE was shown to inhibit human CAII with an IC_50_ value of 17 nM, clearly demonstrating that this inhibitor has a high affinity for the enzyme (Ho *et al*, 2003; Vicker *et al*, 2003). We investigated this hypothesis using three experimental settings: uptake of radiolabelled 667 COUMATE *ex vivo* into rat RBCs, uptake of unlabelled 667 COUMATE *ex vivo* into RBCs and uptake of unlabelled 667 COUMATE *in vivo* in the rat following a single p.o. or i.v. dose. In order to ascertain whether 667 COUMATE is stabilised by the presence of RBCs, we compared the ratio of 667 COUMATE/667 COUMARIN following incubation of the agent with rat plasma or whole blood.

The aims of this study were to determine the pharmacokinetic parameters of 667 COUMATE in the rat and to investigate the hypothesis that this agent is sequestered and stabilised by RBCs. These data will be useful for the design of clinical studies of this STS inhibitor.

## MATERIALS AND METHODS

### Chemicals and reagents

The following reagents were purchased from the suppliers listed: high-performance liquid chromatography (HPLC) grade acetonitrile, diethyl ether (Fisher Scientific UK Limited, Loughborough, Leicestershire, UK); halothane (Astra Zeneca, Cheshire, UK); tetrahydrofuran (THF), acetazolamide, polyethylene glycol-400 (PEG-400), citric acid and citric acid trisodium salt (Sigma-Aldrich Comp. Ltd, Poole, Dorset, UK); water (ICN Biomedicals, Aurora, OH, USA); 0.45 *μ*M HPLC filters (Paul Gelman Laboratories, Portsmouth, UK). 667 COUMATE was prepared as described previously (Woo *et al*, 2000). Radiolabelled 667 COUMATE (30 Ci mmol^−1^) was custom synthesized by Sibtech Inc., CT, USA.

### Pharmacokinetic study

Female Wistar (155–165 g) rats were purchased from Charles River UK Ltd (Margate, Kent, UK) and housed in a dedicated animal facility. Rats received RM1 rodent maintenance diet (SDS, Kent, UK), water *ad libitum* and were maintained in positive pressure isolators under a 12 h light–dark cycle. These experiments were approved by the Imperial College Ethical Review Committee and met the standards required by the UKCCCR guidelines (Workman *et al*, 1998). Rats received 667 COUMATE (10 mg kg^−1^, p.o. or i.v.) with control animals receiving vehicle only (THF : PEG-400: water, 1 : 6 : 3 (in volume)). Three rats were used for each time point. Rats were subjected to terminal anaesthesia (halothane) and blood was removed by cardiac puncture at 2.5, 5, 15, 30 and 45 min and 1, 3, 8, 16 and 24 h after i.v. administration. Following p.o. administration of 667 COUMATE, blood samples were taken at 5, 15, 30 and 45 min and 1, 2, 3, 6, 8, 16, 24 and 48 h. In order to stabilise 667 COUMATE, whole blood was acidified by mixing with one part of 1 M citrate buffer (pH 3) to nine parts of blood. Plasma and RBCs were prepared from whole blood by centrifugation (2800 **g**, 4°C, 15 min). 667 COUMATE and 667 COUMARIN were extracted from plasma (0.5 ml) with diethyl ether (4 ml) and the aqueous phase was frozen in a methanol : solid carbon dioxide mixture. 7-Hydroxycoumarin (3 *μ*g ml^−1^) was used as an internal standard. The organic phase was decanted to a fresh tube and evaporated to dryness under a stream of air at room temperature. The extraction of 667 COUMATE from RBCs used a method that was similar, but not identical, to one developed for the extraction of topotecan from whole blood (Loos *et al*, 2002). Briefly, RBCs were mixed with blank plasma (1 : 1, v v^−1^) and mixed by inversion in order to reconstitute ‘whole blood’. 7-Hydroxycoumarin (3 *μ*g ml^−1^) was added to the whole blood (500 *μ*l). The extraction of the internal standard, 667 COUMATE and 667 COUMARIN into three times the volume of diethyl either was facilitated by vortexing (5 min) and incubating the mixture on ice (10 min). Samples were thawed, vortexed for a further 5 min, centrifuged (15 000 **g**, 15 min) and the supernatant was removed and filtered (0.45 *μ*M, acrodisc CR PTFE). The diethyl ether was removed under a stream of air. The extraction efficiencies for 667 COUMATE from plasma and whole blood were 76±5 and 41±6% (*n*=6), respectively. The residues were stored at −20°C in preparation for HPLC analysis.

### Pharmacokinetic analysis

Pharmacokinetic parameters were calculated using WinNonlin software (Pharsight Corporation, Mountview, CA, USA). The area under the curve (*AUC*) was calculated using the linear trapezoidal method with extrapolation of the terminal phase to infinity. Other parameters calculated were as follows: elimination rate constant (*β*); total body clearance (*Cl*)=dose/*AUC*; volume of distribution (*Vd*)=*Cl*/*β;* elimination half-life (*t*_1/2_*β*)=0.693/*β*; bioavailability (%*F*)=(*AUC*_oral_*/AUC*_i.v._) × 100. Pharmacokinetic data were analysed for differences in plasma 667 COUMATE concentrations after p.o. or i.v. dosing using ANOVA.

### *Ex vivo* investigations into the uptake and stabilisation of 667 COUMATE by RBCs

Whole blood was removed from female Wistar rats, pooled, centrifuged (2800 **g**, 4°C, 15 min) and separated into RBCs and plasma. 667 COUMATE (10 *μ*g ml^−1^), 667 COUMARIN (10 *μ*g ml^−1^) or vehicle only (THF) were added to plasma and mixed with an equivalent volume of RBCs. 667 COUMATE was also incubated with plasma only. Duplicate samples were maintained at 37°C for 0.5, 1, 3 and 24 h on rollers. Whole blood was acidified by addition of 1 M (pH 3) citrate buffer (9 : 1, v v^−1^). In order to determine the extent of 667 COUMATE sequestration by RBCs, plasma and RBCs were separated by centrifugation (2800 **g**, 4°C, 15 min) and reconstituted to whole blood by addition of an equivalent volume of blank RBCs or plasma, respectively. 667 COUMATE and 667 COUMARIN were extracted from ‘whole blood’ as described previously. The effect of the carbonic anhydrase inhibitor acetazolamide (100 *μ*M) on the uptake of 667 COUMATE and 667 COUMARIN into RBCs was also investigated by preincubation with whole blood for 30 min, prior to addition of 667 COUMATE or 667 COUMARIN. For the uptake of [^3^H]667 COUMATE (19 pmol, 1.26 × 10^6^ dpm), the agent was incubated with whole blood (1 ml) for 30 min. Plasma was separated from other blood constituents by centrifugation (2800 **g**, 4°C, 15 min) and the radioactivity was counted.

### High-performance liquid chromatography analysis

667 COUMATE was separated from its putative metabolite 667 COUMARIN using a reversed-phase HPLC method described previously (Ireson *et al*, 2003). Briefly, an Agilent 1100 (Cheshire, UK) autosampler, photodiode array detector, solvent delivery system and C3-phenyl column (250 × 5 mm, 5 *μ*M) purchased from Phenomenex (Cheshire, UK) were used to determine the levels of 667 COUMATE in plasma and whole blood. Plasma calibration curves were found to be linear from 0.1 to 40 *μ*g ml^−1^. The limit of detection of 667 COUMATE in plasma was 0.1 ng ml^−1^. The coefficients of variation (interday and intraday) were <10%.

### Software

Chromatograms were generated using Agilent Chemstation Software. The figures were prepared using Prism version 3 obtained from Graphpad Software, San Diego.

## RESULTS

### Stability of 667 COUMATE in vehicle

To ascertain that 667 COUMATE is stable in the vehicle used, the agent was incubated on ice for up to 6 h. High-performance liquid chromatography analysis of the drug in this vehicle at various time points revealed that 667 COUMATE is stable when maintained under these conditions. 667 COUMARIN, the major degradation product of 667 COUMATE, was below the limit of detection.

### Pharmacokinetics of 667 COUMATE

**Figure 2:**
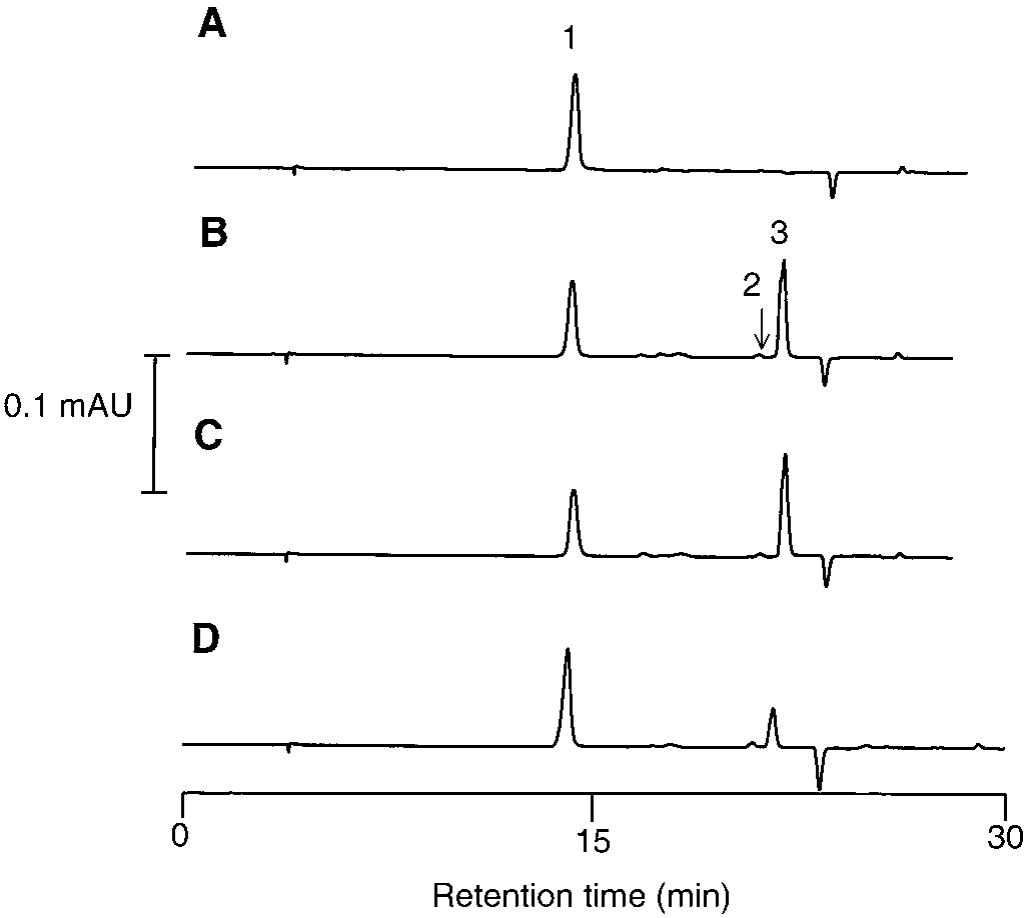
High-performance liquid chromatograms of diethyl ether extracts of rat plasma (**A**–**C**) or RBCs (**D**) 30 min after administration of (**A**) vehicle only, (**B**) 667 COUMATE i.v., (**C**) 667 COUMATE p.o. and (**D**) 667 COUMATE p.o. 7-Hydroxycoumarin, added as the internal standard, 667 COUMARIN and 667 COUMATE are denoted by 1, 2 and 3, respectively. For details of extraction and HPLC method, see Materials and Methods.

In order to determine the pharmacokinetic parameters of 667 COUMATE, plasma concentrations were measured following administration of a single p.o. or i.v. dose to female rats (Figure 2A–D

). 667 COUMARIN, which is generated by degradation of 667 COUMATE in plasma *ex vivo* (Ireson *et al*, 2003), was only a minor metabolic product of 667 COUMATE *in vivo* (Figure 2B and C). A log concentration *vs* time plot showed linear kinetics following both dosing routes. The linear kinetics was indicative of zero order pharmacokinetics, and therefore a noncompartmental model was used for analysis of the data (Table 1

**Table 1:** Summary of 667 COUMATE pharmacokinetic data after 10 mg kg^−1^ bolus i.v. or p.o. administration

**PK parameters**	**i.v.**	**p.o.**
*C*_max_ (*μ*g ml^−1^)	22.8±0.6	11.9±0.4
Elimination rate constant (*β*) (h^−1^)	0.6±0.02	0.3±0.1
*t*_1/2_*β* (h)	1.2±0.1	1.97±0.3
*AUC* (h *μ*g ml^−1^)	35.8±1.3	34.4±1.1
*Vd* (ml kg^−1^)	93.9±2.9	153.2±18.7
*Cl* (ml h^−1^)	53.5±2.6	54.2±2.4
%*F* (bioavailability)	—	95.9±6.1

**Figure 3:**
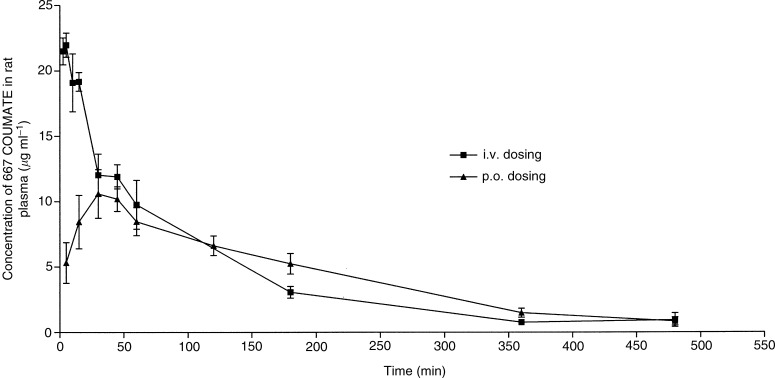
Plasma concentrations of 667 COUMATE after administration of a single p.o. or i.v. dose of 667 COUMATE to female Wistar rats. The values shown in the figure are the means of three animals±s.e.m.

Values shown are mean±s.e.m. (*n*=3).

). 667 COUMATE could be detected for up to 8 h after i.v. and p.o. administration, after which the levels were below the limit of detection (Figure 3

). The maximum level of 667 COUMATE in plasma was attained 30 min after oral dosing. The half-life of 667 COUMATE in plasma was 1.2 and 2 h after i.v. and p.o. dosing, respectively. The bioavailability of 667 COUMATE was approximately 95%. Plasma concentrations of 667 COUMATE were significantly higher 5 and 15 min after i.v. dosing than after p.o. administration (*P*<0.001). At all other time points examined, no significant differences in plasma concentrations resulting from i.v. or p.o. dosing were detected.

### Uptake of 667 COUMATE by RBCs

**Figure 4:**
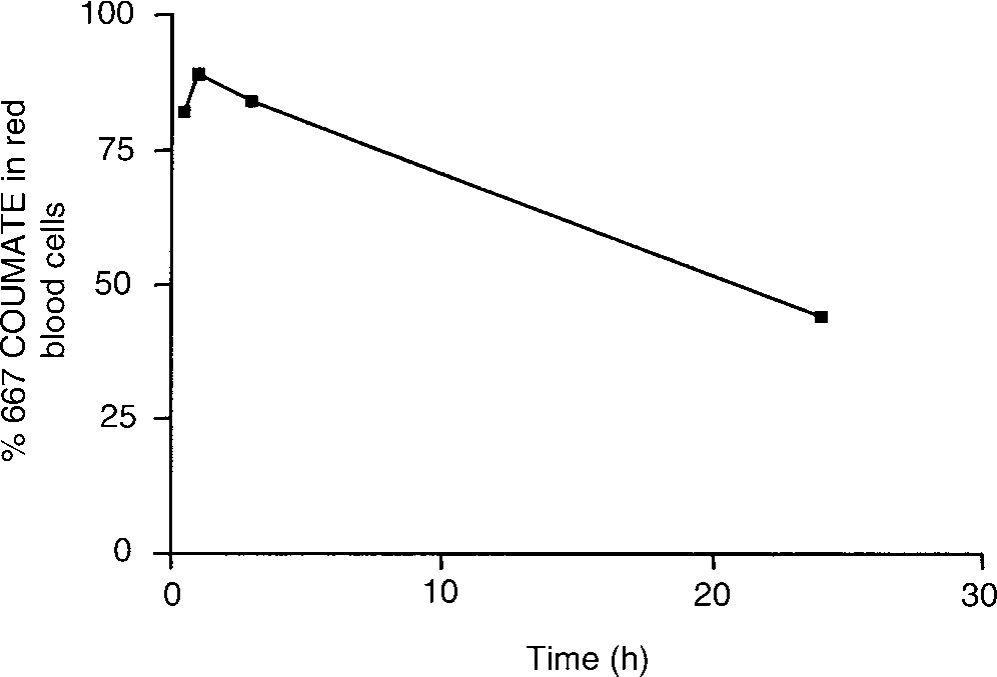
Percentage uptake of 667 COUMATE by rat RBCs following incubation of the agent with whole rat blood maintained at 37°C on rollers. The coefficients of variation between samples were calculated to be less than 10%. For details of extraction of 667 COUMATE from whole blood, see Materials and Methods (means, *n*=6).

We investigated the uptake of 667 COUMATE by RBCs both *ex vivo* and *in vivo.* Following incubation of 667 COUMATE with whole rat blood for 30 min, more than 80% of the agent was found to be associated with the RBC fraction (Figure 4

). This was confirmed by showing that the uptake of radiolabelled 667 COUMATE was also greater than 70%. At 24 h following addition of the drug to whole rat blood, only 48% of the agent was retained by the RBCs. We also established that 667 COUMATE is taken up by RBCs *in vivo* in the rat. The concentrations of 667 COUMATE in RBCs 30 min following administration of the agent p.o. and i.v. were 12 and 19 *μ*g ml^−1^, respectively.

In order to provide evidence that the interaction between CAII and 667 COUMATE is important in the sequestration of the agent by RBCs, whole rat blood was preincubated with the CAII inhibitor acetazolamide. Following incubation with the inhibitor, the uptake of 667 COUMATE into RBCs was reduced to 50%. The uptake of 667 COUMARIN, the degradation product of 667 COUMATE, was 50% and preincubation of blood with acetazolamide did not affect this uptake.

### Stabilisation of 667 COUMATE by RBCs

**Figure 5:**
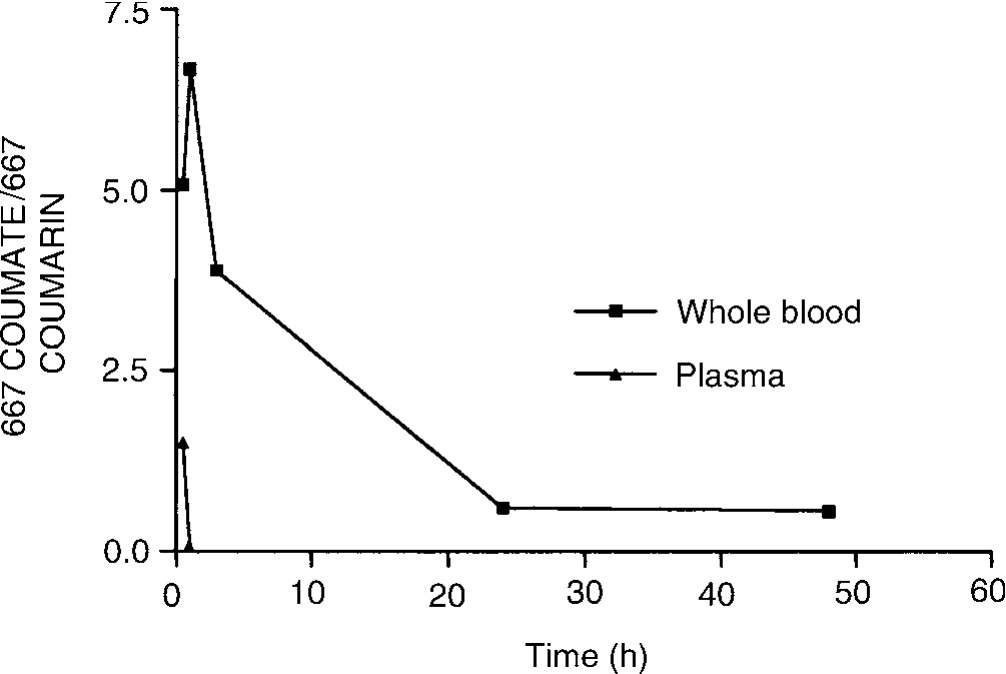
The ratio of 667 COUMATE/667 COUMARIN following incubation of 667 COUMATE (10 *μ*g ml^−1^) with whole blood or plasma. The coefficients of variation between samples were found to be less than 10% (means, *n*=6).

The stability of 667 COUMATE in plasma and whole blood was compared by calculating the ratio of 667 COUMATE/667 COUMARIN at various time points after addition of the agent to these biological fluids. At 30 min after the addition of 667 COUMATE to whole blood and plasma, the ratios of 667 COUMATE : 667 COUMARIN were 5.1 and 1.5, respectively (Figure 5

). When the agent had been incubated with whole blood for 3 h, the ratio was 3.9. In contrast, parent compound 667 COUMATE was below the limit of detection in plasma following a 3 h incubation.

## DISCUSSION

667 COUMATE inhibits rat liver STS by 93% at 10 mg kg^−1^ and causes a reduction in the growth of E1S-stimulated tumours in the nitrosomethylurea-induced rat mammary model (Purohit *et al*, 2000, 2001). The pharmacokinetics of this agent have not previously been investigated. In this study, we elucidated the pharmacokinetic parameters of 667 COUMATE following a single i.v. and p.o. dose of the agent. The clearance rates for 667 COUMATE were 53 and 54 ml h^−1^ following i.v. or p.o. dosing, respectively, suggesting fast removal of 667 COUMATE from the plasma. Such rapid clearance rates would normally indicate that the plasma levels should also fall quickly but this was found not to be the case as significant concentrations of 667 COUMATE were still detectable for up to 8 h. In this study, however, we found that the bioavailability of 667 COUMATE is 95% and that the plasma concentrations of this agent are greater than 0.1 *μ*g ml^−1^ even 8 h after administration of a single p.o. or i.v. dose. 667 COUMATE exhibited a zero order pharmacokinetic profile (Figure 3) when administered via an intravenous route, indicating that it only distributes into one compartment, the blood. However, 667 COUMATE demonstrated a high volume of distribution after oral dosing (153.2 ml kg^−1^) compared to that after dosing i.v. (93.9 ml kg^−1^). This may be due to the difference in the elimination rate constant after oral dosing. The difference in the elimination rate constants may be a consequence of the steady uptake of 667 COUMATE by RBCs following oral absorption, as opposed to the putative rapid saturation of RBC uptake following a bolus i.v. dose, although this remains to be experimentally confirmed. The finding that p.o. administration of 667 COUMATE resulted in similar plasma concentrations to those achieved after i.v. dosing indicates that it will be feasible to give the drug orally when used clinically. It is apparent from this study that 667 COUMATE is relatively resistant to metabolism as no evidence of any conversion to 667 COUMARIN, or other metabolites, was detected. Although tissue concentrations of 667 COUMATE were not measured in the present study, it has been shown to almost completely inhibit STS activity in nitrosomethylurea–induced mammary tumours in rats, indicating that the drug is effectively delivered to target tissues (Purohit *et al*, 2000).

Several studies have demonstrated that RBCs may have a role in the transport of chemotherapeutic agents prominent among which are methotrexate (Lena *et al*, 1987) and 5-fluorouracil (Baerlocher *et al*, 1997). In this study, we set out to determine whether 667 COUMATE is sequestered into RBCs, as this may explain the high bioavailability of this agent despite its relatively high clearance rate. Using two independent analytical methods, we found that when 667 COUMATE is added to rat whole blood *ex vivo*, more than 70% of the agent is taken up by RBCs. We also found that 667 COUMATE is sequestered into RBCs *in vivo*. These findings have stimulated research to identify the site to which 667 COUMATE binds in these cells. It is well known that sulphonamide drugs, such as acetazolamide, which are structurally related to sulphamates through the sulphamido group, can bind to CAII and inhibit its activity (Casini *et al*, 2003). The ability of 667 COUMATE to dock into the CAII active site and inhibit its activity by sulphamate coordination to the enzyme zinc atom was therefore examined (Ho *et al*, 2003; Vicker *et al*, 2003). In addition to being active as an STS inhibitor, 667 COUMATE proved to be also a potent inhibitor of CAII activity in an enzyme preparation derived from RBCs. Evidence from docking studies and CAII inhibition studies suggests that once taken up by RBCs, 667 COUMATE interacts with CAII (Vicker *et al*, 2003). The related steroid sulphamate, EMATE, has been cocrystallised with CAII and the sulphamate–zinc interaction confirmed (Abbate *et al*, 2004). When whole blood was preincubated with the CAII inhibitor acetazolamide, the uptake of 667 COUMATE was reduced to 50%. Consequently, it appears that the interaction of 667 COUMATE with CAII is essential if the agent is to be retained by the RBCs. Studies with other sulphamoylated agents have shown that this interaction is reversible (Ho *et al*, 2003). In this study, we show that 667 COUMATE is above the limit of detection in plasma 8 h after administration of a single i.v. or p.o. bolus of the agent. Furthermore, when the STS inhibitor was incubated with whole blood *ex vivo*, only 48% of the agent was associated with the RBC fraction after 24 h. These data suggest that the interaction of 667 COUMATE with CAII is reversible, although this remains to be experimentally confirmed.

The novel anticancer sulphonamide drug E7070, which is currently undergoing clinical trials, is also taken up by RBCs and this may account for its nonlinear pharmacokinetic profile in humans (Yoshino *et al*, 1999; Terret *et al*, 2003; Van den Bongard *et al*, 2003). Like 667 COUMATE, E7070 is also a potent carbonic anhydrase inhibitor (Supuran, 2003). Expression of carbonic anhydrases is increased in some tumours where their action to acidify the extracellular environment may give tumours a growth advantage over normal tissues (McKierman *et al*, 1997; Tureci *et al*, 1998). Thus, its ability to inhibit carbonic anhydrase activity may contribute to the anticancer efficacy of 667 COUMATE, in addition to its potent STS inhibitory properties.

In our next series of experiments, we investigated the hypothesis that the RBCs provide a sanctuary for 667 COUMATE and protect it from hydrolytic degradation in the plasma. When 667 COUMATE was incubated with plasma, the agent was below the limit of detection after 3 h. In marked contrast, when 667 COUMATE was incubated with whole blood, the agent could still be detected after 48 h. The stabilisation of 667 COUMATE by RBCs may explain why 667 COUMATE, despite its modest stability in plasma, can be detected in plasma for up to 8 h following a single dose and can inhibit hepatic STS *in vivo* in the rat for up to 24 h (Purohit *et al*, 2001). Aryl sulphamates inhibit CAII by binding of the sulphamate moiety to the central zinc ion. Away from this site, the binding site is predominantly hydrophobic and a rigid hydrophobic steroid structure is well accommodated in this site (Abbate *et al*, 2004). 667 COUMATE binds to CAII with an IC_50_ value of 17 nM. As it is a good inhibitor, the more polar coumarin motif must be well accommodated in the hydrophobic site. Since degradation of 667 COUMATE is most likely to be via a *β*-elimination mechanism involving development of charge on the coumarin moiety in the transition state, it seems logical that in a hydrophobic environment, this elimination mechanism is suppressed by this enzyme. This mechanism should be even more favoured here, since sulphamates bind to the CAII zinc as the mono-anion and formation of this mono-anion should stimulate the *β*-elimination process. Crystallisation of 667 COUMATE with CAII is underway and should provide further insight into these proposed mechanisms.

In conclusion, we show that 667 COUMATE is orally bioavailable, despite undergoing rapid degradation in rat plasma. We found that 667 COUMATE is both sequestered and stabilised by RBCs. Therefore, RBCs may protect 667 COUMATE from degradation/metabolic clearance and transport the agent to tissues. These processes may impinge on the pharmacological efficacy of the agent *in vivo* and they support further clinical evaluation of 667 COUMATE as a novel chemotherapeutic agent.

## References

[bib1] Abbate F, Winum J-Y, Potter BVL, Casini A, Montero J-L, Scozzafava A, Supuran CT (2004) Carbonic anhydrase inhibitors: X-ray crystallographic structure of the adduct of human isoenzyme II with EMATE, a dual inhibitor of carbonic anhydrases and steroid sulfatases. Bioorg Med Chem Lett 14: 337–341 14684333 10.1016/j.bmcl.2003.09.064

[bib2] Baerlocher GM, Beer JM, Owen GR, Meiselman HJ, Reinhart WH (1997) The antineoplastic drug 5-fluororacil produces echinocytosis and affects blood rheology. Br J Haemotol 99: 426–433 10.1046/j.1365-2141.1997.4003212.x 9375767

[bib3] Bernstein L, Ross RK (1993) Endogenous hormones and breast cancer. Epidemiol Rev 15: 48–65 8405212 10.1093/oxfordjournals.epirev.a036116

[bib4] Casini A, Antel J, Abbate F, Scozzafava A, David S, Waldeck H, Schafer S, Supuran CT (2003) Carbonic anhydrase inhibitors: SAR and X-ray crystallographic study of the interaction of sugar sulphamates/sulfonamides with isozymes I, II and IV. Bioorg Med Chem Lett 13: 841–845 12617904 10.1016/s0960-894x(03)00029-5

[bib5] Elger W, Palme H-J, Schwarz S (1998) Novel estrogen sulphamates: a new approach to oral hormone therapy. Exp Opin Invest Drugs 7: 575–589 10.1517/13543784.7.4.575 15991994

[bib6] Elger W, Schwarz S, Hedden A, Reddersen B, Schneider B (1995) Sulphamates of various estrogens are prodrugs with increased systemic and reduced hepatic estrogenicity at oral application. J Steroid Biochem Mol Biol 55: 395–403 8541236 10.1016/0960-0760(95)00214-6

[bib7] Howarth NM, Purohit A, Reed MJ, Potter BVL (1994) Oestrone sulphamates: potent inhibitors of oestrone sulfatase with therapeutic potential. J Med Chem 37: 219–221 8295207 10.1021/jm00028a002

[bib8] Ho YT, Purohit A, Vicker N, Newman SP, Robinson JJ, Leese MP, Ganeshapillai D, Woo LWL, Potter BVL, Reed MJ (2003) Inhibition of carbonic anhydrase II by steroidal and non-steroidal sulphamates. Biochem Biophys Res Commun 305: 909–914 12767917 10.1016/s0006-291x(03)00865-9

[bib9] Ireson CR, Parish D, Purohit A, Woo LWL, Potter BVL, Chander SK, Reed MJ (2003) Development of a sensitive high-performance liquid chromatography method for the detection of 667 COUMATE *in vivo*. J Steroid Biochem Mol Biol 84: 337–342 12711020 10.1016/s0960-0760(03)00047-5

[bib10] James MR, Skaar TC, Lee RY, MacPherson A, Zwiebel JA, Ahluwalia BS, Ampy F, Clarke R (2001) Constitutive expression of the steroid sulfatase gene supports the growth of MCF-7 human breast cancer cells *in vitro* and *in vivo*. Endocrinology 142: 1497–1505 11250930 10.1210/endo.142.4.8091

[bib11] James VHT, McNeil JM, Lai LC, Newton CJ, Ghilchik MW, Reed MJ (1987) Aromatase activity in normal breast and breast tumor tissue: *in vivo* and *in vitro* studies. Steroids 50: 269–279 3509763 10.1016/0039-128x(83)90077-6

[bib12] James VHT, Reed MJ (1980) Steroid hormones and human cancer. Prog Cancer Res Ther 14: 471–487

[bib13] Lena N, Imbert AM, Brunet P, Cano JP, Carcassonne Y (1987) Kinetics of methotrexate and its metabolites in red blood cells. Cancer Drug Deliv 4: 119–127 2448023 10.1089/cdd.1987.4.119

[bib14] Lin JH, Lin TH, Cheng H (1992) Uptake and stereoselective binding of the enantiomers of MK-927, a potent carbonic anhydrase inhibitor, by human erythrocytes *in vitro*. Pharmaceutical Sci 9: 339–344 10.1023/a:1015886717974 1614967

[bib15] Loos WJ, van Zomeren DM, Gekderblom H, Verweij J, Nooter K, Stoter G, Sparreboom A (2002) Determination of topotecan in human whole blood and unwashed erythrocytes by high-performance liquid chromatography. J Chromatogr 766: 99–105 10.1016/s0378-4347(01)00432-7 11820300

[bib16] McKierman JM, Buttyan R, Stifelman MD, Kutz AG, Chen MW, Olsson CA, Sawczuk IS (1997) Expression of tumor-associated gene MN: a potential biomarker of human renal cell carcinoma. Cancer Res 57: 2362–2365 9192809

[bib17] Miyoshi Y, Ando A, Hsegawa S, Ishitobi M, Aaguchi T, Tamaki Y, Noguchi S (2003) High expression of steroid sulfatase mRNA predicts poor prognosis in patients with estrogen receptor-positive breast cancer. Clin Cancer Res 9: 2288–2293 12796397

[bib18] Noel CT, Reed MJ, Jacobs HS, James VHT (1981) The plasma concentration of oestrone sulphate in postmenopausal women: lack of diurnal variation, effect of ovariectomy, age and weight. J Steroid Biochem 14: 1101–1105 7198170 10.1016/0022-4731(81)90039-x

[bib19] Parkin DM, Pisani P, Ferlay J (1999) Estimates of the worldwide incidence of 25 major cancers. Int J Cancer 80: 827–841 10074914 10.1002/(sici)1097-0215(19990315)80:6<827::aid-ijc6>3.0.co;2-p

[bib20] Pasqualini JR, Chetrite G, Blacker C, Feinstein MC, Delalonde M, Talbi M, Maloche C (1996) Concentrations of estrone, estradiol and estrone sulfate and evaluation of sulfatase and aromatase activities in pre- and postmenopausal breast cancer patients. J Clin Endocrinol Metab 81: 1460–1464 8636351 10.1210/jcem.81.4.8636351

[bib21] Purohit A, Woo LWL, Barrow D, Hejaz HAM, Nicholson RI, Potter BVL, Reed M.J (2001) Non-steroidal and steroidal sulphamates: new drugs for cancer therapy. Mol Cell Endocrinol 171: 129–135 11165021 10.1016/s0303-7207(00)00428-7

[bib22] Purohit A, Woo LWL, Potter BVL, Reed MJ (2000) *In vivo* inhibition of estrone sulfatase activity and growth of nitrosomethylurea-induced mammary tumors by 667 COUMATE. Cancer Res 60: 3394–3396 10910045

[bib23] Reed MJ, Purohit A, Woo LWL, Potter BVL (1996) The development of steroid sulphatase inhibitors. Endocr Relat Cancer 3: 9–23

[bib24] Strott CA (2002) Sulfonation and molecular action. Endocr Rev 23: 703–732 12372849 10.1210/er.2001-0040

[bib25] Supuran CT (2003) Indisulam: an anticancer sulphonamide in clinical development. Expert Opin Invest Drugs 12: 283–287 10.1517/13543784.12.2.283 12556221

[bib26] Suzuki T, Nakata T, Miki Y, Kaneko C, Moriya T, Ishida T, Akinaga S, Hirakara H, Kimura M, Sasano H (2003) Estrogen sulfotransferase and steroid sulfatase in human breast carcinoma. Cancer Res 3: 2762–2770 12782580

[bib27] Terret C, Zanetta S, Roche H, Schellens JH, Faber MN, Wanders J, Ravie M, Droz JP, EORTC Early Clinical Study Group (2003) Phase I clinical and pharmacokinetic study of E7070, a novel sulphonamide given as a 5-day continuous infusion repeated every 3 weeks in patients with solid tumours. Eur J Cancer 39: 1097–1109 12736109 10.1016/s0959-8049(03)00128-x

[bib28] Tureci O, Sahin E, Vollmar E, Siemer S, Gottert E, Seutz G, Parkila A-K, Shah GN, Grubb HJ, Pfeundschuh M, Sly WS (1998) Human carbonic anhydrase XII: cDNA cloning, expression and chromosomal localization of a carbonic anhydrase gene that is overexpressed in some renal cancers. Proc Natl Acad Sci USA 95: 7608–7613 9636197 10.1073/pnas.95.13.7608 PMC22698

[bib29] Utsumi T, Yoshimura N, Takeuchi S, Ando J, Maruta M, Maeda K, Harada N (1999) Steroid sulfatase expression is an independent predictor of recurrence in human breast cancer. Cancer Res 59: 141–145 9927050

[bib30] Van den Bongard HJGD, Pluim D, Van Waardenburg RCAM, Ravic M, Beijnen JH, Schellens JHM (2003) *In vitro* pharmacokinetic study of the novel anticancer agent E7070: red blood cell and plasma protein binding in human blood. Anti-Cancer Drugs 14: 405–410 12853880 10.1097/00001813-200307000-00003

[bib31] Vicker NJ, Ho YT, Robinson JJ, Woo LWL, Purohit A, Reed MJ, Potter BVL (2003) Docking studies of sulphamate inhibitors of oestrone sulphatase in human carbonic anhydrase *II*. Bioorg Med Chem Lett 13: 863–865 12617909 10.1016/s0960-894x(03)00009-x

[bib32] Woo LWL, Purohit A, Malini B, Reed MJ, Potter BVL (2000) Potent active site-directed inhibition of steroid sulphatase by tricyclic coumarin-based sulphamates. Chem Biol 7: 773–791 11033081 10.1016/s1074-5521(00)00023-5

[bib33] Workman P, Twentyman P, Balkwill F, Balmain A, Chaplin D, Double J, Embleton J, Newell D, Raymond R, Stables J, Stephens T, Wallace J (1998) United Kingdom Co-ordinating Committee on Cancer Research (UKCCCR) guidelines for the welfare of animals in experimental neoplasia (second edition). Br J Cancer 77: 1–10 10.1038/bjc.1998.1 PMC2151254 9459138

[bib34] Yoshimura N, Harada N, Bukholm I, Karesen R, Borresen-Dale A-L, Kristensen VN (2004) Intratumoral mRNA expression of genes from the oestradiol metabolic pathway and clinical and histopathological parameters of breast cancer. Breast Cancer Res 6: R46–R55 14979917 10.1186/bcr746 PMC400649

[bib35] Yoshino OT, Okauchi H, Yoshimatsu T, Ozawa K, Sugi Y, Nagasu NH, Koutanngi T, Kitoh K (1999) Discovery of novel antitumor sulfonamides targeting G1 phase of the cell cycle. J Med Chem 42: 3789–3799 10508428 10.1021/jm9902638

